# Neuroimmune multi-hit perspective of coronaviral infection

**DOI:** 10.1186/s12974-021-02282-0

**Published:** 2021-10-13

**Authors:** Shawn Hayley, Hongyu Sun

**Affiliations:** Department of Neuroscience, Carleton University, 1125 Colonel By Drive, Ottawa, ON K1S 5B6 Canada

**Keywords:** COVID19, Neurodegeneration, Microglia, Stress, Viral, ACE2

## Abstract

It is well accepted that environmental stressors experienced over a one’s life, from microbial infections to chemical toxicants to even psychological stressors, ultimately shape central nervous system (CNS) functioning but can also contribute to its eventual breakdown. The severity, timing and type of such environmental “hits”, woven together with genetic factors, likely determine what CNS outcomes become apparent. This focused review assesses the current COVID-19 pandemic through the lens of a multi-hit framework and disuses how the SARS-COV-2 virus (causative agent) might impact the brain and potentially interact with other environmental insults. What the long-term consequences of SAR2 COV-2 upon neuronal processes is yet unclear, but emerging evidence is suggesting the possibility of microglial or other inflammatory factors as potentially contributing to neurodegenerative illnesses. Finally, it is critical to consider the impact of the virus in the context of the substantial psychosocial stress that has been associated with the global pandemic. Indeed, the loneliness, fear to the future and loss of social support alone has exerted a massive impact upon individuals, especially the vulnerable very young and the elderly. The substantial upswing in depression, anxiety and eating disorders is evidence of this and in the years to come, this might be matched by a similar spike in dementia, as well as motor and cognitive neurodegenerative diseases.

## Background

Sophisticated defensive strategies have evolved to protect the sensitive neuronal cells and their circuitry from environmental xenobiotic and pathogenic threats. This includes not only the obvious blockades provided by the blood brain barrier (BBB) and the blood cerebrospinal barrier, but also the specialized cells of the immune system [71]. The brain’s specialized immunocompetent cell, the microglia, plays a major role in this regard, along with important support from astroglial cells [61, 73]. Although tightly regulated, the entry of peripheral immune cells, including macrophages, neutrophils, NK cells and T and B lymphocytes, can also occur and this process is greatly upregulated during times of cellular distress or microbial invasion [108, 127]. These processes generally result in neutralization or elimination of the threat and may result in certain cases, in long-term protection. However, there is also the possibility that such exposures might prime inflammatory cascades, such that the magnitude of future threats might be exaggerated. This idea of a sensitization of neuroinflammatory cascades has been applied to the understanding of how neurodegenerative disease might evolve over time with repeated environmental stressor exposures [8, 79]. There is also the thought that the distress caused by combined pathogenic along with xenobiotic “hits” might simply act to cumulatively add up to exceed some threshold that will eventually result in pathology ensuing [46, 110, 113]. The nature of pathology that develops with repeated environmental hits of course, depends upon one’s genetic constitution and what “weak links” a person possess. Ultimately however, it is important to consider how parallel changes in peripheral immunity and organ systems interact the central nervous system (CNS) in the context of repeated xenobiotic and pathogenic threats.

At the heart of most neurological diseases, there is a some degree of infiltration of peripheral inflammatory immune cells, along with activation of local glial cells [12, 20, 68, 97, 108]. Accordingly, animal models of  neurodegenerative conditions, such as Alzheimer’s and Parkinson’s disease (PD), are characterized by a substantial neuroinflammatory component [7, 21, 27]. Moreover, administration of immunogenic challenges, such as high doses of lipopolysaccharide (LPS), murine hepatitis virus (MHV) or polyinosinic:polycytidylic acid (poly I:C), can induce marked activation of microglial cells and astrocytes and in some cases lead to peripheral immune cell infiltration or even neuronal death, coupled with behavioral deficits [8, 79, 84, 136]. Intriguingly, even neuropsychiatric disorders, such as depression, anxiety and schizophrenia, have also been linked to an over-activation of the inflammatory immune system, albeit without obvious neuronal loss [1, 2, 5, 53, 67]. In fact, even psychological stressors, if sufficient, can stimulate immune cell mobilization and the release of soluble inflammatory factors, including a variety of cytokines [34, 100].

With the current coronavirus disease (COVID19) pandemic underway and its far-reaching global impact, it is of particular importance to consider the long-term psychiatric and neurological consequences that will ensue over the next few years, as well as the possible ripple effects on brain health that still might be felt decades later. Individuals can be impacted by the virus itself or from the secondary stress associated with it, including the fearfulness, loneliness and loss of social ties. Consistent with the theme of this review, the COVID19 pandemic itself may be thought of comprising a series of “hits” (including the pathogenic effects of the virus itself or the isolation, loneliness and sense of lack of control that characterizes the psychosocial aspects of pandemic) that collectively can impact neuroimmune functioning. Such “pandemic hits” would be expected to have differential effects on individuals, depending upon what environmental or stressful insults that have already been experienced over one’s lifetime. Of course, the impact of future threats or stresses would likewise be shaped by one’s stressor history. It is also of upmost importance to bear in mind that microbial agents, such as those of the coronavirus severe acute respiratory syndrome (SARS) family which cause COVID19, can impact multiple biological systems and the inter-connections between these different systems might be crucially alerted. Indeed, in the current pandemic there have been reported a number of co-morbidities, ranging from gastrointestinal, depressive symptoms, olfactory disturbances and cognitive difficulties [10, 24, 64, 131]. This review seeks to explore the impact of the coronavirus and the pandemic conditions upon the neuroimmune system and place in context, the potential mechanisms through which viral invasion might interact with other environmental threats or individual vulnerabilities to influence long-term CNS outcomes.

## Main text

### Neural–immune co-evolution: a physiological arms race

It is believed that some of the earliest cellular creatures on earth possess some rudimentary immune-like apparatus that allowed for defence and maintenance of homeostasis. This would include bacteria, viruses and other parasites that would have existed during such ancient times. Hence, the earliest sensing mechanisms that allows for extracellular interactions likely would have been immune based. Essentially, the immune system would act to detect and provide protection from various stressors. This would have placed co-evolutionary pressure on evolving multicellular organisms and single-celled pathogens to develop more sophisticated and “sneaky” detection apparatus over time. At the same time, it could have also paved the wave for more complex sensing strategies that allowed for communication between multicellular organisms. Such grouping behaviors or “cell social” aggregations could endow further benefits against potential pathogens and would require substantial energetic input and sophisticated structures.

Over time, highly complex organisms evolved further sensing capacities that went beyond typical immune based pathogenic threat detection and developed more general threat detection systems. It is conceivable that such evolving sophisticated systems tapped into and elaborated upon existing immune and CNS processes. Indeed, evidence of the co-evolution of the CNS and immune systems is suggested based on the relatively high number of brain antigens that have been implicated in autoimmune disease. To this end, enzymes or receptors for the neurotransmitters GABA (GAD65) and acetylcholine (AchR), as well as myelin, laminin and tyrosine hydroxylase are targeted in several autoimmune diseases, including diabetes, psoriasis, multiple sclerosis and rheumatoid arthritis [92]. It is curious that only a small subset of the relatively limitless number possible antigens are responsible for such autoimmune diseases and that these tend to be found in abundance in the CNS. Further still, it is intriguing to note that autoimmunity linked to tumors or infectious agents disproportionately targets brain antigens, such as myelin [92]. Hence, there is reason to believe that the CNS might house many such “super-autoantigens” capable of overcoming the normal inhibitory restraints of the immune system. It is also conceivable that this might be linked to the evolutionary pressures that shaped bi-directional brain–immune cooperation. In effect, such antigens might be vestiges from past mechanisms for immune protection of the brain or even as a means to foster synaptic plasticity and/or coordinate widespread nascent neural circuits.

The co-evolution of brain and immune systems has also been driven, at least in part, by our increasing social links and construction of wide-ranging communities. This has concomitantly fostered the development of more sophisticated CNS networks, but at the same time a greater potential spread of pathogens. This later point of course, has been presumably met by increasing immunological defensive strategies, but also substantially complex potential disease possibilities. On top of microbial threats, the modern psychosocial world has opened the door to novel stressors, such as unmet social needs that can give rise to loneliness and sense alienation. The current pandemic would push the boundaries and exacerbate such conditions. Interestingly, a recent imaging study even went as far as demonstrating a unique loneliness-linked neural signature that included a series of brain regions known as the default network [124]. These higher associated brain regions together are critical for our sense of self and consciousness, but may also provide introspection on social events that are lacking and could ultimately, result in rumination and inner distress. Of course, this could have important implications for defining how pandemic-related loneliness and general psychosocial stress might prompt depressive and maladaptive or obsessive behavioral patterns.

Modern immune-related sensing and appraisal systems would eventually also become linked with stress regulatory hormonal (HPA) and neurochemical (autonomic) systems, along with brain mechanisms for detecting threat and organizing social behaviors. Certainly, much work has demonstrated that the immune system, particularly through inflammatory cytokines, can markedly impact CNS stress circuitry, along with hormonal and neurochemical outcomes [29, 76]. Some brain regions impacted by cytokines include, the amygdala, which is critically involved in threat appraisal and the hippocampus, cingulate and prefrontal cortex [18, 65] that all have critical roles in shaping social behavior and developing highly plastic learning designed to maximize survival.

Recent evidence is emerging to confirm a role for the immune system in the regulation of highly complex social processes. It makes intuitive sense that the immune system and brain would co-evolve as master regulators of organismic responses to external stimuli with a goal of maintaining internal homeostasis. It also stands to reason that these systems are so highly intertwined in a bi-directional manner in order to facilitate a dialogue aimed at optimally positioning the organism within the environment. With increasing interactions with conspecifics leading to highly complex social interactions among individuals, there also is an added likelihood of increased spread of pathogenic organisms [81]. This could help fuel the concomitant evolution of further specialized immune mechanisms to meet the progressive expansion of social links. Also, with increased sociability comes greater emotional attachments that aid in survival. However, increased emotionally opens the door to a range of new sensing problems, such as when attachments become compromised (as in the case of the isolation provoked by the current global pandemic) and psychosocial stress ensues and emotional pathology can result, such as the case with depression and anxiety-related disorders.

The relatively newly evolved adaptive immune system may play a particularly germane role in both normal social functioning, as well as in cases of psychosocial stress. Indeed, T lymphocyte deficient severe combined immunodeficiency (SCID) mice were reported to have deficits in social preference and this deficit was reversed upon repopulation with wild-type lymphocytes [31]. It was further discovered that T lymphocytes found in the cerebrospinal fluid (CSF) secreted the pro-inflammatory cytokine, IFN-γ, and that his cytokine was most important for the organization of social behaviors. In fact, evidence from rodents, fruit flies and zebrafish all found that social conditions influenced IFN-γ-responsive genes, with an upregulation in the face of social interactions, but a reduction when social isolation occurred [31]. Further still, variations in adaptive immune cells were reported among wild mammals to be affected by social living conditions, as well as age and sex. For example, it was reported that variations in lymphocyte proportions (in males but not females) among wild European badgers was influenced by social group size and age, being smaller and declining faster with age in those animals living in relatively small groups, compared to those living in larger social groups [74].

Other pro-inflammatory cytokines, such as TNF-α, may also play a critical role in the regulation of complex social behaviors, through their ability to modify synaptic plasticity in hippocampal and cortical circuits. Indeed, TNF-α activates glutamatergic synaptic transmission by increasing presynaptic activity, postsynaptic AMPAR trafficking and synaptic insertion, while at the same time, suppressing inhibitory synaptic transmission [41, 125]. Further, TNF-α has been implicated in homeostatic (non-Hebbian) synaptic scaling, through its ability to regulate the quantity of synaptic AMPA receptors, as well as by synaptic pruning by controlling the phagocytosis capacity of microglia [47, 125]. Similarly, the multi-protein complex, called the inflammasome, that controls the secretion of the pro-inflammatory cytokines, IL-1β and IL-18, is also thought to regulate synaptic pruning and play a role in neuronal survival [134]. It appears that IL-1β, in turn, modulates intracellular Ca^2+^ predominately through NMDA receptors in a dose-dependent manner. At physiological concentrations, IL-1β is able to increase Ca^2+^ influx and enhance Hebbian-based LTP, while pathophysiological levels of IL-1β decreased Ca^2+^ influx and impaired LTP [104, 109]. Ultimately, it seems that the pro-inflammatory cytokines may act as soluble mediators of neural plasticity and affect circuits that could be important for social processes.

From an evolutionary perspective, pathogens, such as SARS-COV2, have long been competing and hence, co-evolving, with both immunological and CNS processes. Thus, immunological defences (both innate and adaptive) and brain defences (e.g., BBB) have evolved parallel sophisticated anti-pathogen strategies. At the same time, the pathogens themselves rather quickly (largely owing to their high genetic turnover rates) have evolved means of evading these defensive strategies. These strategies often revolve around the up- and down-regulation of specific receptors or increasing affinity for new targets, or the induction of some other structural changes [22, 58]. Other tricky strategies might include the release of soluble inhibitory factors or peptides with immunomodulatory properties [98]. Whatever the case, the eternal embrace for which the pathogen-detection systems are engaged in represent a remarkable endless biological struggle.

### Viral peripheral and central immunity

Viral agents are first detected by innate immune cells bearing pattern recognition receptors (PRRs), which detect distinct evolutionarily conserved structures on pathogens, termed pathogen-associated molecular patterns. Among these, the toll-like receptors (TLRs), are particularly important and are expressed on CNS microglia regulating their detection of pathogenic threats [37, 52]. Intracellular viral RNA, including that of SARS-COV2, is generally detected by TLR3, whereas the spike proteins from the virus can be recognized by TLRs 1, 4 and 6 [17]. However, SARS-COV2 primarily enters and infects cells by first binding to the angiotensin converting enzyme 2 (ACE2) membrane protein. Indeed, the spike protein of coronaviruses SARS-COV binds to ACE2 and facilitates its entry into cells. Hence, TLR-linked inflammatory signaling appears likely to occur only after ACE2 has been engaged by the virus, in an attempt to contain viral replication and spread. It has also been reported that other coronavirus proteins, such as ORF3a, can provoke NLRP3 inflammasome activity [49], which can further perpetuate neuroinflammatory cascades and accumulation of various peptides.

To add further complexity to the mix, there is the possibility of “sterile inflammation” or the induction of an inflammatory cascade in the absence of a direct antigenic/pathogenic threat. Indeed, some of the same receptor systems that detect pathogen threats can also be trigger by non-immune danger-associated molecular patterns (DAMPs) when cells are sufficiently distressed. In particular, in cases of tissue damage (such as stroke or head injury) DAMPs are released from damaged cells [40]. Chronic stressor exposure (in the form of electric shock and noise) in rodents likewise provoked the DAMP, HMGB1 and its receptor RAGE, and this effect was observed specifically in microglial cells [135]. This is yet another ingenious strategy, wherein non-pathogenic stressors are able to “detected or sensed” by a common defensive innate immune process. Hence, it is important to appreciate that during times of stress, these danger signals may further exacerbate any ongoing inflammatory responses, such as those provoked by infectious or other microbial challenges.

It is generally thought that SARS-COV-2 invasion of the brain occurs either through a hematogenous (from peripheral immune cells) route or alternatively, possibly by migrating by way of an olfactory neural route [87, 93]. It is also likely that the virus itself can disrupt the BBB integrity, though its induction of systemic inflammation and that this can further augment neuroinvasion [112]. Since deficits in olfaction and taste are commonly reported during early stages of COVID19 [49], it was suggested that coronaviruses may be able to travel into the CNS through retrograde axonal transport via the cribriform plate [25]. Viral invasion of the CNS might also occur by way of retrograde synaptic transport via axons from receptors in the lung into the respiratory areas within the medulla of the brainstem [72] or of course, through circulatory or lymphatic routes [9, 75]. Once within the brain, SARS-COV2 infection may have neuroimmune effects either by, (a) direct entry into the intracellular compartment of neurons or glia, or by (b) inducing secondary damage from systemically or locally derived inflammatory cells or soluble factors.

Some critical recent evidence questions whether the SARS-COV2 virus actually penetrates the brain parenchyma, but rather suggests that CNS effects might stem from the transmission of inflammatory signals from the choroid plexus at the level of the blood–cerebrospinal fluid barrier [132]. In fact, Yang et al. [132] found that gene expression profiles from the choroid plexus and medial prefrontal cortex of individuals that died from COVID19 failed to detect virus RNA or protein, but did reveal marked changes in numerous inflammatory genes.

Studies from early in the COVID19 pandemic indicated that approximately 37% of severe cases that required hospitalization had neurological symptoms and these patients also had especially high levels of pro-inflammatory cytokines and other innate inflammatory factors, including C-reactive protein (CRP) [69, 72, 80]. The most common symptoms reported were confusion, seizures, headache, dizziness, impaired consciousness, gait deficits, cerebrovascular pathology and encephalitis [91]. Pathological peripheral symptomology also includes deficits in cranial nerve functioning that can give rise to compromised smell and taste, or the manifestation of elements of autoimmune disease, such as Guillain–Barré syndrome. Of course, the caveat must be mentioned that a great number of the more serious and hospitalized patients were also individuals of advanced age and this alone, is often associated with a greater inflammatory tone. In a sense, this might represent a sort of “perfect storm”, wherein the already existing potential age-dependent peripheral and central inflammatory state (along with any pre-existing disease) might interact with the viral hit resulting in exaggerated collateral damage within the CNS. This is consistent with the present theme of a multi-hit phenomenon promoting a disturbed neuroimmune phenotype.

### Multi-hit concept as applied to SARS2-COV2

Diseases ranging from cancer to Parkinson’s to depression have been framed according to their link to multiple environmental insults over the course of one’s lifetime. In all these cases, it is likely the cumulative effects of differing environmental insults over time that eventually elicits illness. It could be envisioned as a tipping point being reached and the break point is likely determined by genetic vulnerabilities. The timing between these individual “hits” is probably just as important as how many and the severity of hits. Indeed, in the case of immune processes (and neural ones as well for that matter) the timing between hits is critical in shaping their impact. For instance, we and others have found that the time between exposure to differing immune insults, such as TNF-α, IL-1β, LPS, poly I:C, or between these insults and differing stressors (restraint, foot shock, chronic mild stress) or toxins (paraquat, MPTP) greatly influenced the nature of various outcomes observed, including motor behavior, sickness symptoms, stress hormonal levels, neurotransmitter turnover and even the degree of neurodegeneration evident [8, 38, 45, 59, 79, 105]. Thus, it is possible that time-locked neuro-immune-related sensitizing and/or synergistic processes are a fairly universal phenomenon, applicable to a range of environmental insults.

If one views COVID19 as an environmental hit (and a very large one for many individuals) then it follows that many individuals may develop a long-term vulnerability to subsequent stressors or environmental insults. Further, when one considers the multi-system impact of the virus and associated stressor sequelae then the neuroimmune impact might be substantial. The cytokine storm that can occur in COVID19 is a particularly salient aspect of the disease that could reasonably be expected to elicit marked responses across many bodily systems. Indeed, the pro-inflammatory cytokines induced are extremely potent and require rapid buffering to restrain their potentially damaging consequences across multiple organ systems. If unchecked, these cytokines can cause tissue dysregulation and entrenched biological changes that can have enduring consequences. Within the CNS, these cytokines can affect neurogenesis, dendritic structural plasticity, mitochondrial functioning, synaptic neurotransmission, synaptic plasticity and overall cellular homeostasis. Prolonged excessive levels can be catastrophic for neurons and lead to hyper-active glial responses.

Timing might be very important when considering the impact of immune factors in the context of coronaviral infection. It is already known that the kinetics of the antibody and T cell response following SARS-COV2 infection might be different among patients and vary as a function of disease severity [66, 90]. Also, the initial immune response to the viral infection is sometimes followed by a secondary hyper-active inflammatory response in many individuals, which can be especially toxic to organ functioning [86]. It is not yet clear whether responses of innate immune cells, such as macrophages or the brain’s microglia might also retain some sort of time-linked “memory” in response to the virus. We do know that these innate cells robustly respond to environmental toxins and some theories of CNS disease, suggest that they might become “locked” into a hyper-activated state over time, especially in vulnerable elderly individuals [43, 50].

### Multi-hit concept as applied to SARS2-COV2: inflammatory and microglial priming

**Fig. 1:**
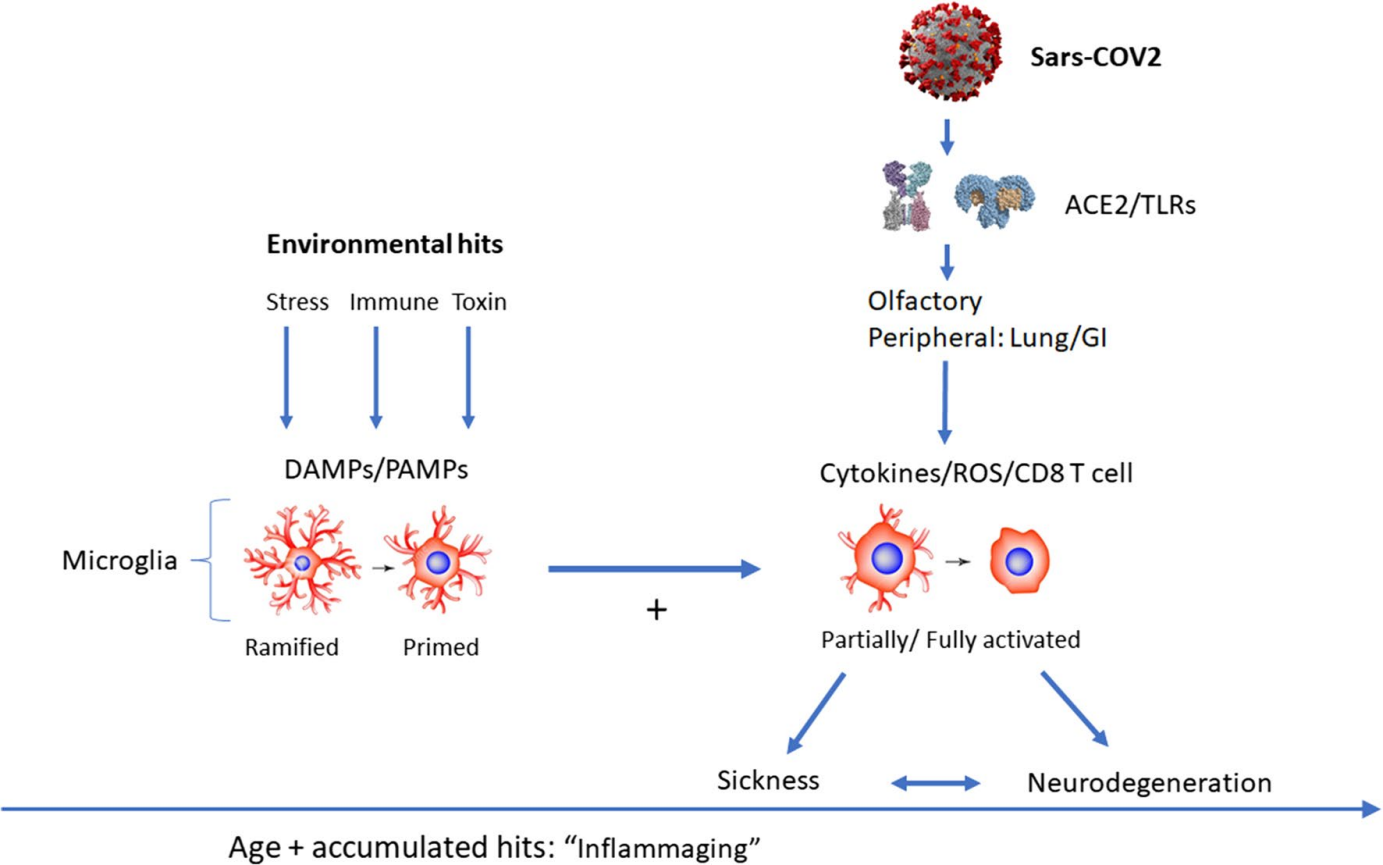
Multiple environmental hits may prime microglia to modify responsiveness to SARS-COV2 and enhance the impact upon the central nervous system (CNS). Microglial cells can become “primed” as a result of advancing age and accumulated exposure to stressors, immune insults and potential environmental toxins. All of these insults have the ability to induce pathogen-associated molecular pattern (PAMPs) and damage/danger-associated molecular pattern (DAMPs) that act upon microglia and potentially induce a state of inflammatory aging or “Inflammaging”. Subsequent exposure to the SARS-COV2 virus may then further stimulate microglia and favor an “activated” pro-inflammatory phenotype. The virus, acting through ACE2 proteins and TLR receptors, may either directly enter the CNS via olfactory nerves or indirectly act by stimulating peripheral targets in the lung, gastrointestinal or other organs. Infected CD8+ T lymphocytes appear able (albeit in a limited capacity) to enter the CNS or alternatively, soluble cytokines and other inflammatory and oxidative stress factors might secondarily impact the brain. Ultimately, such factors can interact with local microglia to orchestrate a neuroinflammatory milieu, which can impact neurons and favor sickness symptoms or in extreme situations (and with chronic activation) might contribute to neuronal pathology or conceivably, even neurodegeneration in the long run

The concept of sensitization may be especially relevant when considering SARS-COV2 as a ‘hit’ that might have and enduring impact upon the brain. Indeed, as depicted in Fig. 1, age-dependent exposure to various environmental hits can lead to “inflammaging” and a priming of microglia, which may be further exacerbated by exposure to SARS-COV2. Such a primed microglial state might then engender further vulnerability and the likelihood of sickness responses emerging and even, over time, neurodegeneration. It is in fact well known that immunological events can sensitize subsequent biological responses. In fact, many allergies or autoimmune disorders can develop over time as a result of sensitization following exposure to various antigens in susceptible individuals. While these responses involve adaptive immune memory cells (T and B lymphocytes), there is also evidence that innate branches of immunity, involving macrophages and microglia, might also impart some degree of plasticity and “memory” for insults. Indeed, innate immune memory might take the form of either sensitization or tolerance, wherein enhanced or suppressed immune responses to a secondary insult are apparent. Such responses may be provoked by the same or differing insults upon subsequent exposures [19, 123, 94].

Paralleling the peripheral immune memory responses, central priming of microglial cells with immune challenges can lead to either an augmented (sensitized) or diminished (tolerance) responses to subsequent challenges. In the most severe immune sensitized cases, wherein systemic inflammation causes sepsis or septic shock sets in, unregulated inflammatory cytokine release and BBB breakdown occurs that can fuel microglial sensitivity. Such a situation involves a so-called “cytokine storm”, which is a term that does not really have a particularly clear definition but rather generally refers to a collection of pro-inflammatory cytokines, notably IL-1, IL-6, TNF-α and IFN-β, that are released in abnormally high concentrations. If unchecked such peripheral inflammatory factors can adversely impact the brain. For example, in a mouse model of sepsis (cecal ligation and perforation) there was a shift in microglia to an amoeboid morphological profile with increased pro-inflammatory cytokines evident in the hippocampus [123]. These mice were also more susceptible to synapse damage in response to subtoxic amounts of b-amyloid, which stemmed from increased microglial phagocytic activity against hippocampal synapses [123].

Much recent attention has been devoted to the fact that some COVID19 patients displayed evidence of such a cytokine storm, with elevations in IL-2, G-CSF, IL-7, TNF-α along with the chemokines, IP-10, MCP-1, MIP-1A [121]. Likewise, a recent paper found IL-6 to be markedly elevated among SARS-COV2 infected patients and in fact, disease severity was correlated with circulating levels of this cytokine [42]. Yet, others report that the cytokine storm is likely not a major factor in widespread pathology in SARS-COV2 patients [120]. Nonetheless, excessive cytokine responses might have particularly dramatic consequences in the “long run” on elderly individuals that already have some degree of age-related inflammatory pathology or “inflamm-aging”. This may be one reason why elderly individuals are much more prone to the cytokine-mediated severe organ pathology. Indeed, the excessive inflamm-aging is already associated with elevated systemic pro-inflammatory cytokine levels, as well as a reduction in anti-inflammatory mechanisms that normally keep such processes in check [85]. By the same token, inflammatory aging is also characterized by excessive reactive oxygen factors, reductions in protective autophagic processes, as well as alterations in ACE2 expression that impact viral spread [85]. Hence, all roads during aging may lead to the same increase in inflammatory cytokine-associated damage.

With regard to the brain and especially relevant from a multi-hit perspective, a cross-sensitization between the immune system and brain might occur, wherein neuro-immune communication is disturbed. As an example, a single injection of the cytokines, IL-1β or TNF-α, provoked a sensitized neurochemical state, such that later exposure to these cytokines or to stressors evoked exaggerated behavioral, hormonal and central monoamine responses [44, 116]. But timing again is important here, TNF-α pre-treatment followed by re-exposure to the same cytokine 2–4 weeks later resulted in a very exaggerated HPA and behavioral sickness responses, however, changes in neurotransmission in cortical and extra-hypothalamic brain regions tended to be larger when re-exposure to the cytokine occurred after earlier intervals of 1–7 days [44]. Indeed, TNF-α re-exposure after longer intervals induced widespread illness reminiscent of systemic shock that was coupled with marked hypothalamic neurochemical alterations [3]. However, TNF-α re-exposure after briefly intervals (with presumably less time to sensitize adaptive immunity) prompted neurochemical changes within the prefrontal cortex in the absence of systemic illness [44, 45]. Such time-linked responses hint at the possibility that infectious events that markedly elevate cytokines might similarly be able to differentially modulate CNS processes, depending upon their temporal proximity to other environmental insults.

Similar to their ability to time-dependently affect neural signaling, there is experimental evidence to suggest that immunological insults might also sensitize processes that can influence neurodegeneration. In this regard, pre-treatment of mice with either viral (poly I:C) or bacterial (LPS) agents time-dependently augmented the neurodegenerative response elicited by later toxin (the neurotoxic pesticide, paraquat) exposure [8, 80]. Importantly, the enhanced neurodegenerative response was associated with microglial priming, such that the impact of the second hit (paraquat) was augmented when it coincided with a time when microglia displayed an “activated” morphology. This occurred 2 (at peak microglial response) but not after 7 days following the initial LPS or poly I:C priming stimulus [8, 80].

It remains to be determined what factors might control microglial priming, but it has been suggested that various cytokines or inflammatory factors could play a role and these might be particularly apparent in the degenerating or injured brain [102]. It also might be the case that augmented expression of Fc or other immune receptors might be up-regulated on primed microglia allowing their interactions with infiltrating adaptive immune cells or antibodies in the case of disease-related blood brain barrier breakdown [102]. There is also evidence that priming efficacy might be influenced by both developmental stage and dose of the eliciting stimulus. Indeed, when priming with very low doses of LPS, microglia derived from newborn rodents displayed enhanced pro-inflammatory cytokines and iNOS and augmented BNDF expression compared to those obtained from adult or aged murine brains [70]. In contrast, the administration of much higher doses of LPS resulted in a tolerance effect, wherein reduced pro-inflammatory cytokines, iNOS and BDNF was evident and this occurred regardless of the developmental state [70].

Genetic analysis studies have begun to focus upon the collection of factors that might be responsible for the differing primed states of microglia. It was found that mice primed with a high LPS dose of LPS (10 mg/kg), displayed a very characteristic molecular profile that was enriched with genes critical for lysosomal and phagosome functioning, along with those associated with antigen presentation [94]. In particular, primed microglial genes included Axl, Apoe, Clec7a, Itgax, and Lgals3, which were referred to as a “disease-associated microglia (DAM) signature” [63]. At the same time, these same authors found many genes involved in homeostatic regulation were diminished with microglial priming, these included Cx3cr1, P2ry12 and Tmem119. Further studies have uncovered evidence of epigenetic mechanisms underlying microglial priming. In this regard, epigenetic silencing of the Il1b promoter in microglia was evident in mice following low-dose LPS (0.25 mg/kg) priming and this resulted in diminished CNS IL-1b levels in response to a second challenge with the endotoxin 32 weeks later [114]. Hence, epigenetic reprogramming might play a role in shaping a specific DAM “signature” that has long-term consequences on microglial reactivity and it is conceivable that such microglial signature priming might also occur in the context of SARS-COV2 or other infectious agents.

### Multi-hit concept as applied to SARS2-COV2: aging and neurodegeneration

The process of aging alone over one’s lifetime may result in a gradual shift towards a primed neuroinflammatory microglial state. Curiously, this somewhat differs from the peripheral immune counterpart, the macrophage, which tends to display age-accumulated senescence and diminished inflammatory responses [107]. In this regard, microbial (e.g., LPS) or traumatic (e.g., surgery) insults were reported to provoke blunted peripheral macrophage-driven inflammatory responses in aged rodents [6]. Intriguingly however, the opposite effect was observed within the CNS, wherein enhanced microglial-driven neuroinflammatory responses were observed in aged mice [39, 54]. Hence, divergent innate inflammatory responses may occur in the CNS vs periphery in aged animals. Although the reason behind this is unclear, it is conceivable that the delicate CNS tissue may require more robust defensive responses against microbial threats and the overall microbial/toxin load that might accumulate in the body over long periods of time. Finally, the density and reactivity of microglia appears to vary between differing brain regions, being influenced by varied CNS microenvironments. Accordingly, age-dependent variations in microglial state are not uniform across brain regions, just as the case that age-dependent neuronal damage/degeneration is very much CNS location specific.

The aging process, together with an individual’s immune/stressor history may promote low-grade inflammation and an “inflammaging” phenotype [19]. Such a persisting inflammatory background has been posited to be able to prime individuals to the development of various age-related diseases, as well as sensitize them to the impact of subsequent infectious agents (Fig. 1) [96]. Intriguingly, it was posited that innate inflammation that has beneficial consequences earlier in life, might switch to being detrimental with advanced age (possibly owing to the diminished evolutionary pressure presumed at advanced ages) [33]. Of course, not only microbial factors, but trauma or tissue damage that sometimes occurs with the aging process, such as with serious reductions in cerebral blood flow may promote inflammatory priming. Ischemic stroke did induce marked cytokine changes and increase protein levels of ACE2 in the lungs (and presumably aid viral infiltration), which correlated with the severity of behavioral deficits that occurred [119]. Ultimately, it be may be that a “pro-inflammatory threshold” exists that when exceeded gives rise to age-related illnesses, such as various cancers and neurodegenerative disease [33].

There is the question as to whether individuals already with existing CNS disorders might be especially vulnerable to COVID19 and if they do develop the disease, would this impact their primary disease state. For instance, might COVID19 increase psychiatric or neurological symptoms in individuals already suffering from depression, anxiety, Alzheimer’s or  Parkinson’s disease (PD)? It is already known that there are a higher proportion of hospitalized COVID19 patients that also have a neurodegenerative disease and that these individuals have a particularly high mortality rate [30, 88]. Yet, it is unclear as to whether the infection affects the trajectory of the underlying neurodegenerative disease, as this would require future longitudinal studies among survivors. Similarly, while it is safe to say that the stress associated with COVID19 might precipitate depressive or anxious pathology, it will be of interest to determine whether the immune sequelae per se induced by the virus can do the same. Since neuro-immune links are inherently bi-directional, the cytokines or other factors at play in COVID19 could certainly be expected to disturb this delicate communication and potentially dysregulate both peripheral and CNS elements of disease.

The multi-hit hypothesis has received particular attention regarding the origins of neurodegenerative diseases, most notably PD, wherein the majority of cases are idiopathic and believed to involve some degree of environmental triggers. Further still, the idea of multiple microbes interacting may acts as “hits” to exert some degree cumulative damage might contribute to PD development [28]. Indeed, one study found that PD patients were more likely to have been infected with multiple pathogens, compared to age-matched controls and these pathogens included *Helicobacter pylori*, Epstein Barr virus, *Chlamydia pneumoniae* and herpes simplex virus [13]. Further evidence for a multi-hit idea comes from the general finding of a lack of robust pathological phenotype in rodents expressing various PD implicated genes. Rather an additional factor, such as an immunological insult is required to provoke behavioral and functional pathology reminiscent clinical disease [95]. For instance, mice bearing the common PD-linked mutation, G2019S LRRK2, had exacerbated dopamine neuron loss and motor impairment following exposure to the toxicant, MPTP, compared to those expressing the wild-type LRRK2 gene [4, 60]. Similarly, treatment of G2019S LRRK2 mice with a single dose of the common experimental endotoxin, LPS, provoked exaggerated neuroinflammatory changes in the cortex and ventral brain regions with PET imaging of [11C] PBR28, compared to non-transgenic mice [115]. Other studies likewise revealed increased inflammatory (but not neurodegenerative) outcomes in G2019S LRRK2 mutants treated with LPS together with the toxicant, paraquat [26].

Similar to PD, some evidence points to the possibility that multiple toxins and genetic mutations might collectively contribute to late-onset Alzheimer’s disease (LOAD), whereas in contrast, the early onset form of disease appears to be largely determined by single gene variants [111]. In effect, it was postulated that LOAD may occur slowly over time resulting from progressive homeostatic imbalances that stem from inefficient energy metabolism. Such shifts in energy homeostasis would leave individuals with an inability to compensate for the impact of accumulating environmental insults [111]. Similarly, it has been suggested that the build-up of soluble Aβ oligomers might act as an initial step [35], that can render individuals vulnerable to subsequent insults that conceivably, occur in the context of slowly unfolding time-dependent genetic programs.

Just as is the case for other neurodegenerative disorders, there is reason to believe that microglial priming might occur with aging and repeated environmental hits and potentially lead to the development of LOAD. It was indeed posited that infectious or other hits during critical brain periods lead to microglial “priming”, which could contribute to the development of disease [51]. Furthermore, acute and chronic LPS administration differentially influenced various outcomes in APP23 Alzheimer’s transgenic mice. For instance, at 6 months following an individual acute LPS injection in APP23 mice, there were diminished anti-inflammatory IL-10 levels and increased Aβ plaque accumulation [130]; whereas, a more chronic LPS schedule produced an opposing outcome with a reduction of pro-inflammatory IL-1b levels, coupled with decreased plaque load [130]. Whatever the case, it remains to be determined whether or not SARS-COV2 exposure might act as an important “hit” that primes microglial (either towards a sensitized or tolerant phenotype) and if so, what are the long-term implications with regard to CNS disease outcomes.

In addition to neurodegeneration, SARS-COV2 may provoke neuropsychiatric illnesses, either alone or as a co-morbid feature of another disease. One study that assessed over 400 COVID-19 survivors found evidence for various psychiatric symptoms [83]. In this regard, around 30% of patients self-reported signs of depression and about 40% complained of excessive anxiety and insomnia. Collectively, 56% these individuals scored in the pathological range and these scores correlated with measures of systemic inflammation (lymphocyte, neutrophil, and platelet counts [83]). Another recent article has even advocated for the repurposing of tetracycline drugs, that can inhibit microglial reactivity and neuroinflammation, for treating COVID19-related depressive illness [15]. Indeed, there has been a plethora of research implicating inflammatory processes in general and microglia in particular, in the genesis of clinical depression [128, 133]. Hence, it has been suggested COVID-19 survivors be periodically assessed with regard to potential inflammatory biomarkers that might foster the emergence of psychiatric symptoms [83].

### Coronaviral long-term impact on the brain

Time will tell what possible long-term consequences of SARS-COV2 upon the brain will emerge. In the case of the 1918 influenza pandemic, we know that a sizable number of survivors developed acute encephalitis, and some went on to develop clear neurological syndromes, involving motor or cognitive deficits [32, 126]. While it might only be a small number of COVID19 individuals that suffer actual neuronal damage, there may be a sizable number that face “low grade” inflammatory processes that contribute vulnerability and may affect healthy “brain ageing”. Of course, another factor is the stress of the social isolation and constant fear over one’s health that undoubtedly is taking a toll in large populations throughout the world. The rate of psychiatric illness is already on the rise and will likely continue to do so. For many, COVID19 might be the “last straw” or give rise to conditions that act as a tipping point for mental illness.

Aging has emerged as a huge key risk factor for the pathological impact of SARS-COV2, with mortality influenced by age above virtually all other factors. It remains to be determined how age will modulate long-term brain functioning in the face of COVID19 disease, but we do certainly know that microglia in the aged brain are more likely to produce exaggerated or at least somewhat abnormal neuroinflammatory cascades in response to subsequent challenges. As mentioned earlier, the excessive inflammatory aging (Inflamm-Aging) that is often present in many elderly individuals could result in a subgroup of COVID patients (ones that survive the acute effects of the infection) developing further CNS complications and possible earlier death in the years following the infection.

Post-mortem assessment of the brains of patients that died from COVID19 have revealed several abnormalities, consistent with neuropathology. These include edema, neuronal loss, necrosis, glial hyperplasia and signs of ischemia and demyelination [62, 99, 118]. In particular, collateral damage has been reported such as ischemic strokes, stemming from cerebrovascular abnormalities produced by the viral spread and subsequent inflammatory stress upon the cardiovascular system [48, 89]. Many such neuropathological features have been, more or less, recapitulated in mice inoculated with various coronaviruses. For instance, infection with the coronavirus, hCoV-OC43, produced spongiform-like degeneration in mice, along with impaired motor activity and poor limb clasping [56, 55]. Alarmingly, behavioral effects were evident months following viral infection and neuronal degeneration within the hippocampus was detected after one full year. The authors found that even following clearance of the virus, its viral RNA still persisted for several months. It was also speculated that following recovery from the prominent clinical symptoms, there still may be a low level of persisting virus present. In a sense, a low-grade or latent infection could conceivably persist and exert cumulative long-term effects. It could also potentially interact with other microbial threats or environmental stressors yielding additive or synergistic outcomes on a variety of neural or glial systems. We have previously found that cytokine induced synergistic effects are in fact, frequently observed with regard to neurotransmission, as well as stress hormonal and behavioral changes following combined exposure to LPS, poly I:C and stressors [11, 36].

Encephalopathy has been reported in a number of SARS-CoV-2-infected cases and is often in association with delirium, agitation or motor disturbances. These clinical symptoms are generally found in the context of increased peripheral inflammatory factors, but surprisingly, typically normal levels were apparent within the CSF [48]. Similarly, there was a notable absence of SARS-COV2 viral fragments detected in CSF from severe neurological cases, even in those showing EEG abnormalities [23, 48]. Yet, other emerging findings indicate that SARS-COV2 does have some affinity for the brain and this is particularly evident in severe cases. One such study reported that about 36% of patients with severe COVID infections (hospitalized in Wuhan) had neurological symptoms [80] and an even more striking parallel study reported this number to be as high as 90% in cases in a hospital in France [48]. A similarly conducted German study found SARS-COV2 RNA in 53% of the brains of fatal COVID-19 cases [82].

A couple of recent intriguing studies, using human brain organoids, presented evidence that SARS-COV2 viral particles could infect and replicate within neurons, eventually potentially inducing neuronal death [101, 106, 135]. Yet this neuronal death did not co-localize directly with virus infection, but there were metabolic changes in the infected cells. Hence, it was posited that the infected cells might provoke disturbances that favor hypoxic or other injury to neighboring cells [122]. Further, the fact that anti-viral antibodies were also recently reported in the CSF of a neurologically ill COVID-19 patient [129], raises the possibility that the adaptive immune response to the virus might penetrate the brain to modify neurological status.

Ongoing studies are confirming that ACE2 is likely expressed in both CNS neurons and glial cells [16]. Intriguingly, ACE2 levels appear to be brain-region specific being particularly high in the olfactory bulb and in pericytes associated with the BBB, but also were detected in the hippocampus, substantia nigra, middle temporal gyrus, and posterior cingulate cortex [16]. Another intriguing recent study using post-mortem human tissue found ACE2 expression throughout the brain, with the amygdala, cerebral cortex and brainstem showing the most robust expression [77]. Hence, it appears likely that the SARS-COV2 virus has the capacity to infiltrate the brain and have the capacity to act directly to cause neuropathology under some circumstances.

With regard to microglia, recent post-mortem tissue from those that died from COVID19 provide evidence to suggest that at least some brain microglia are in an “activated” phenotype and that they co-exist with infiltrating CD8+ T cytotoxic cells (particularly in the medulla brain region) [82]. It was further suggested that brain microglia likely play a role in stimulating the invading CD8+ T cells, thereby priming these peripherally derived cells within the CNS. This would suggest localized brain immunity and antigen presentation can occur. Interestingly, there were not really any signs that frank neurodegeneration was occurring in these patients, suggesting that, at least in the short run, any CNS primed cells did not show a proclivity towards neurotoxicity. This is not to say that neuronal health was not affected and that over time, there may eventually be some degree of neuronal pathology or degeneration.

Although a small number of SARS-COV2 viral particles were localized in the brain [82], these might have come about through infection or alternatively, stem from BBB weakening as a result of the severity of disease. Yet, it is interesting that the microglia and CD8+ T cell findings were very consistent and did not vary as a function of the patient co-morbidities (all had some of co-morbid disease, most notably cardiovascular and pulmonary disease, or cancers) or rapidity of disease spread. Overall, it seems that the brain is not an immediate SARS-COV2 target in terms of gross neural pathology and that it will take some time before we know the actual long-term consequences of the virus.

Even in the absence of actual viral penetration into the brain, there are emerging data suggesting that peripheral immune cells and soluble factors might enter the CNS. For instance, a just released comprehensive post-mortem study has provided more compelling evidence of infiltrating CD8+ T cells and microglial activation following severe COVID19 disease [117]. In this study, COVID19 patients had significant CD8+ T cell infiltration, together with signs of activated CD68 and the TMEM119 expressing microglia, compared to controls and those that died from non-COVID19-related respiratory illness. Overall, 80% of COVID19 patients had increased levels of either diffuse or dense clusters (microglial nodes) of microglia, 68% displayed detectable CD8+ T cell infiltration into the parenchyma and 36%–44% had the most severe phenotype, which was associated with some degree of axonal damage (but no signs of necrotic cell death) in the medulla [117].

Finally, immune T cell infiltration and microglial activation appears to vary between anatomical regions. Indeed, the infiltration by T lymphocytes and activation of microglia was particularly evident within most the brainstem and cerebellum, and fresh ischemic lesions were also observed in a subset of these patients [82]. A further post-mortem study by Poloni et al. [103] detected prominent CD68-positive microglia that were again, most apparent in the brainstem of COVID-19 cases (this was in contrast to the higher microglia levels in the hippocampus and frontal cortex of a comparison group of Alzheimer’s patients). It was also observed that sparse pockets of T lymphocytes appeared to infiltrate the CNS, wherein they tended to cluster around microglial-rich regions. Yet, the fact that there was little presence of the actual SARS-COV2 virus within the brain [103], suggests that the virus does not have to actually breach CNS barriers in order to exert central immunity and influence delicate neural circuitry. Taken together, the emerging evidence seems to suggest that post-COVID-19 brains are characterized by a prominent innate microglial response, with some signs of T lymphocyte-driven adaptive immunity, but more subtle changes in neuronal integrity and no obvious signs of encephalitis or frank neurodegeneration. Although, it should be considered that there is a possibility that the virus might exacerbate or sensitize neurons to the acute effects of cerebral stroke, given the increase in ischemic lesions in COVID19 patients [82]. According to a “multi-hit” framework, with the passage of time and increasing age (and further environmental hits) there may emerge neuronal damage/degeneration in post-COVID-19 patients, but only further longitudinal studies will determine what future outcomes will resemble.

## Conclusions

The rapidly emerging data indicate that SARS-COV2 provokes a highly complex range of symptoms that can impact multiple systems, including the CNS. While the profile of individuals that develop serious disease is heterogenous, it is absolutely clear that advanced age is a predominate risk factor. It also seems evident that age-related changes in immunity and inflammatory processes in particular, are critically linked to disease severity. Of course, a number of individuals will ultimately succumb to the disease, but among the survivors, it is posited that there may be various sub-groups that go on to develop neurological illness as a result of the viral exposure acting in concert with any other environmental stressors. It is possible that the acute very marked upregulation of cytokines (so-called cytokine storm) that follows serious pulmonary respiratory disease might have long-term sensitizing effects that could lead to further disease. Likewise, it is also possible that chronic low-grade inflammation might persist following resolution of the viral infection and this could subsequently interact with further stressors to spur disease. Of course, beyond the biological impact of the actual viral infection, the concomitant psychological distress in the form of worry, loneliness, sense of helplessness and loss of social supports should also be considered. Such psychogenic stressors can have potent effects that would act as a further “hit’ in the context of our multi-hit model. Whatever the case, longitudinal studies will be required to address such questions.

The development of novel immunotherapeutics to combat viral infection must critically be evaluated, so as to not adversely interact with neuroimmune elements. For instance, it would be important to take steps to avoid antibody-dependent toxicity. In this case, IgG antibodies that bind to viral antigens (e.g., spike glycoprotein S1), could conceivably, induce neuroinflammation through their infiltration into the host immune cells via their Fc receptors. In fact, there has been at least one observation of a paradoxical rise in disease severity in the presence of viral-directed antibody titers [14]. Yet, there is scant evidence to support a direct role for S1-IgG triggered activation of the complement system and subsequent pathology. But the N protein of SARS-CoV-2 was shown to stimulate the complement system, resulting in elevated C3 and C5a complement proteins in the lungs and serum [57]. It was further suggested that activated complement elements can damage sensitive CNS cells, following their activation either peripherally or within the confines of cerebroventricular system [78].

Research inspired by the COVID19 pandemic will undoubtedly open new doors to better understanding micro-processes of cellular immunity and emerging viruses, all the way up to macro-processes of globalization and health care delivery. The heterogeneity of COVID19 symptoms, including those involving the CNS, has reinforced the connectedness to the brain and immune system and underscored the importance of considering the delicate orchestration between such systems. Perhaps above all else, we have learned that science-driven global cooperation across research and medical issues is critical for preventing further catastrophes.

## Data Availability

Not applicable.

## Abbreviations

ACE2: Angiotensin-converting enzyme 2
AMPA: α-Amino-3-hydroxy-5-methyl-4-isoxazolepropionic acid receptor
Arp 2/3: Actin-related protein 2/3 complex
BBB: Blood brain barrier
CD209: Cluster of differentiation 209
CNS: Central nervous system
COVID19: Coronavirus disease 2019
CRP: C-reactive protein
CSF: Cerebrospinal fluid
DAMP: Damage-associated molecular pattern
hCoV-OC43: Human coronavirus OC43
HPA: Hypothalamic pituitary adrenal
IFN-g: Interferon- γ
IL-1b: Interleukin-1b
IL-6: Interleukin-6
IP10: Interferon γ-induced protein 10
LOAD: Late-onset Alzheimer’s disease
LPS: Lipopolysaccharide
LRRK2: Leucine-rich repeat kinase 2
LTP: Long-term potentiation
MCP-1: Monocyte chemoattractant protein-1
MHV: Murine hepatitis virus
MIP1A: Macrophage inflammatory protein 1
MPTP: 1-Methyl-4-phenyl-1,2,3,6-tetrahydropyridine
NK cells: Natural killer cells
NMDA: N-Methyl-D-aspartate receptor
PD: Parkinson’s disease
Poly I:C: Polyinosinic:polycytidylic acid
SARS-COV2: Severe acute respiratory syndrome coronavirus 2
SCID: Severe combined immunodeficiency disease
TLR: Toll-like receptor
TMPRSS2: Transmembrane protease, serine2
TNF-a: Tumor necrosis factor-α
